# Exploring Contraindicated Medications and Corresponding Targeted Genes for Migraine Through Integrated Genetic Approaches

**DOI:** 10.1002/brb3.71056

**Published:** 2025-11-19

**Authors:** Nan Wang, Weixuan Liang, Zhuofeng Wen, Wanzhe Liao, Zhixin Xie, Zhiyi Zhou, Ziyang Yang, Xitong Ju, Haobin Zhou, Chuiguo Huang

**Affiliations:** ^1^ Department of Anesthesiology Nanjing Jiangning Hospital Nanjing China; ^2^ The First School of Clinical Medicine Guangzhou Medical University Guangzhou China; ^3^ The Sixth School of Clinical Medicine Guangzhou Medical University Guangzhou China; ^4^ Nanshan School Guangzhou Medical University Guangzhou China; ^5^ The Second School of Clinical Medicine Guangzhou Medical University Guangzhou China; ^6^ Third Clinical College of Guangzhou Medical University Guangzhou China; ^7^ Institute of Clinical Pharmacology Anhui Medical University Hefei Anhui China; ^8^ Department of Medicine and Therapeutics, Prince of Wales Hospital The Chinese University of Hong Kong Hong Kong

**Keywords:** contraindicated drugs, genetic, migraine, migraine with aura, target gene

## Abstract

**Objective:**

To identify contraindicated medications and corresponding target genes for migraine and its subtypes.

**Method:**

Utilizing the Genome‐Wide Association Studies (GWAS) for 14 medication‐use categories from UK Biobank and GWAS for migraine and its subtypes from FinnGen, our study revealed potential contraindicated drugs for the chronic paroxysmal neurological disorder based on Mendelian randomization (MR). Then we applied the Transcriptome‐wide association study (TWAS) within the framework of Omnibus Transcriptome Test using Expression Reference Summary data (OTTERS) and cross‐referenced three drug databases to determine the targeted genes of the identified contraindicated medications. Moreover, Summary‐data‐based Mendelian Randomization (SMR) and INtegration of TWAS And ColocalizaTion (INTACT) were used as sensitivity analyses to further validate the associations and strengthen causal inference. We also performed brain‐tissue TWAS across 13 regions to confirm the tissue‐specific associations of the hub genes with migraine and its subtypes.

**Results:**

MR analysis revealed that the diuretics and agents that act on the renin‐angiotensin system were discovered to be associated with an increased risk of migraine and migraine with aura. By intersecting the results from TWAS and drug databases, 37 and 22 target genes of contraindicated medications were identified for migraine and migraine with aura, respectively. SMR and INTACT confirmed the validity of *BLM, C12orf76, GPATCH4, PAM, SERPINC1* for migraine, and *ALMS1, GPATCH4, NCF2, SLC12A1, ZKSCAN8P1* for migraine with aura. At the brain‐tissue level, we further validated hub gene associations: in migraine, SERPINC1, ZKSCAN8P1, C12orf76, and PAM were significant across brain regions; in migraine with aura, SERPINC1, ALMS1, ZKSCAN8P1, PAM, and NCF2 were significant.

**Conclusion:**

We identified the diuretics and agents that act on the renin‐angiotensin system as the contraindicated medications for migraine and migraine with aura, and *BLM, C12orf76, GPATCH4, PAM, SERPINC* were found as the targeted genes of contraindicated drugs for migraine and *ALMS1, GPATCH4, NCF2, SLC12A1, ZKSCAN8P1* for migraine with aura, providing new clues for the preventive strategies for migraine and its subtypes.

## Graphical abstract

To systematically identify risk medications for migraine and its subtypes, we integrated GWAS data for 23 medications with GWASs of migraine and its subtypes to conduct causal inference. We then combined plasma eQTLs with drug‐target databases to map putative targets of the risk medications and validated causal relationships using colocalization and SMR analyses. In total, we identified six drug‐target genes associated with migraine risk and six associated with migraine with aura, with their associations replicated across 13 brain regions.

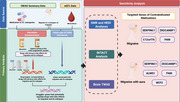

## 1 Introduction

Migraine is a chronic paroxysmal neurological disorder characterized by multiphase attacks of head pain and a myriad of neurological symptoms (Dodick 2018). The latest findings from the Global Burden of Disease Study highlight that migraine, the second leading cause of disability worldwide and the leading cause among young women, affects 18% of women and 6% of men globally (Steiner et al. 2020; Goadsby et al. 2017). However, due to the involvement of various factors, managing migraine presents significant challenges, and medications are a major trigger for migraine (Oliveira et al. 2024; May and Schulte 2016). Nitroglycerin is widely recognized as a drug that induces migraines, likely through the activation of the nitric oxide‐cGMP pathway (Thomsen et al. 1994). Additionally, a recent study suggests that reserpine may influence migraine attacks by regulating serotonin levels in platelets (Genefke et al. 1975), and hydralazine and ranitidine have also been reported to cause migraine symptoms (Silberstein 2004). In addition to these specific medications, previous research has also identified inappropriate medication behaviors, such as overuse of acute migraine medications and ineffective acute treatments, as significant factors contributing to migraine occurrence, while research on contraindicated medications for migraine, which refers to the drugs with adverse side effect triggering or intensifying migraine, and their associated risk mechanisms is still limited (May and Schulte 2016; Stone et al. 2016; Hovaguimian and Roth 2022). Therefore, there is an urgent need to study these contraindicated drugs and their mechanisms of action to improve the clinical prevention of migraines (Jackson et al. 2015; Ha and Gonzalez 2019).

Among the current methods for revealing the relationship between drugs and diseases, Mendelian randomization (MR) is particularly effective. It leverages Genome‐wide association studies (GWAS) data and sets specific genetic variants as instrumental variables (IVs) to infer causal relationships between drugs and diseases, thereby minimizing confounding and addressing reverse causality. For instance, Bowen Tang and colleagues have utilized MR to uncover the relationship between antidiabetic drugs and the risk of Alzheimer's disease (Tang et al. 2022). Additionally, Transcriptome‐Wide Association Studies (TWAS) offer a means to identify genes influencing complex traits and diseases, overcoming the lack of research on the risk mechanisms of contraindicated medications (Mancuso et al. 2017; Gusev et al. 2018; Mancuso et al. 2018; Wainberg et al. 2019; Strunz et al. 2020). Each of these approaches has its own merits, thus we tried to synthesize the strengths of these approaches to form an integrated and multi‐layered genomic approach, for better exploring the reliable contraindicated drugs and drug targets.

**FIGURE 1:**
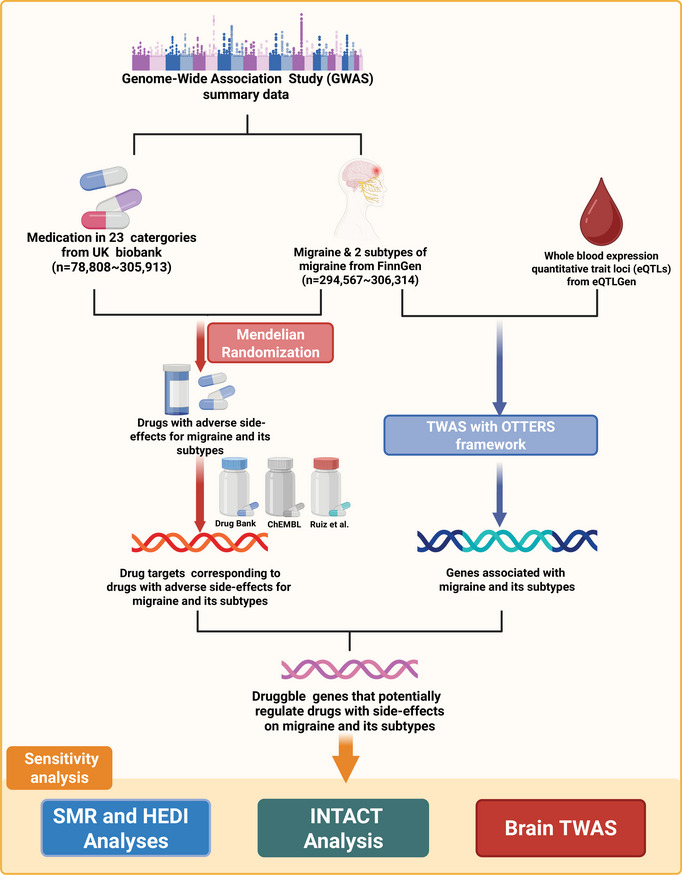
Schematic diagram of the study framework.

We initially included GWAS summary statistics for 23 medication‐used categories to begin our systematic screening process. After excluding medication categories without valid instrumental variables and those directly related to migraine, 14 of them remained. We then conducted a MR analysis using the GWAS summary statistics for these 14 medication‐use categories along with those for migraine and its two subtypes to identify contraindicated drugs. Subsequently, we applied TWAS within the Omnibus Transcriptome Test using Expression Reference Summary data (OTTERS) framework to identify genes related to migraine. We retrieved the target genes of contraindicated medications for migraine and its subtypes from three drug databases, DrugBank (Wishart et al. 2018), Ruiz et al. (2021), and ChEMBL (Gaulton et al. 2017), and intersected these with the target genes identified in TWAS. Furthermore, we performed Summary‐data‐based Mendelian Randomization (SMR) and INtegration of TWAS And ColocalizaTion (INTACT) as sensitivity analyses to further substantiate causal relationships. Finally, we applied brain‐tissue TWAS to validate the associations of hub genes with migraine and migraine with aura at the brain‐tissue level. Figure 1 presents the analytical framework that comprehensively underpinned the study.

## 2 Method

### 2.1 Data Source

#### 2.1.1 GWAS Data for Medication‐Used Categories

Contraindicated medications for migraine and its subtypes are defined as drugs with adverse side effects on migraine, which trigger or worsen the migraine and its subtypes. Thus, we obtained the GWAS summary statistics for 23 medication‐use categories from the study by Yeda Wu et al., which included up to 502,616 participants from the UK Biobank (UKB), with approximately 54% of the cohort being female (Wu et al. 2019). The size of the dataset varied across different medication categories, ranging from 78,808 to 305,913 participants, as detailed in Table S1.

#### 2.1.2 GWAS Data for Migraine and Subtypes of Migraine

GWAS summary statistics for migraines and its two subtypes, including migraine with aura and migraine without aura, from the FinnGen consortium were utilized (Kurki et al. 2023). The data encompassed 18,477 cases and 287,837 controls for migraine overall, 7917 cases and 287,837 controls for migraine with aura, and 6730 cases and 287,837 controls for migraine without aura, as detailed in Table S1.

#### 2.1.3 eQTL (Expression Quantitative Trait Loci) Data

Our research derived the cis‐eQTL (within ±1 megabase of gene transcription start sites) summary‐level data for 16,699 genes from eQTLGen Consortium for subsequent analyses, which features a meta‐analysis of 37 cohorts with a total of 31,684 samples (Vosa et al. 2021). The dataset primarily consisted of blood samples (25,482 samples, accounting for 80.4%) and peripheral blood mononuclear cell samples (6202 samples, accounting for 19.6%). The brain eQTL pre‐trained weights were derived from Functional Summary based Imputation (FUSION) (Gusev et al. 2016) and cover 13 regions: Amygdala, Anterior cingulate cortex (BA24), Caudate (basal ganglia), Cerebellar Hemisphere, Cerebellum, Cortex, Frontal Cortex (BA9), Hippocampus, Hypothalamus, Nucleus accumbens (basal ganglia), Putamen (basal ganglia), Spinal cord (cervical C‐1), and Substantia nigra. Sample sizes range from 100 to 188, as detailed in Table S1.

### 2.2 Mendelian Randomization

To identify contraindicated medications for migraine with its two subtypes, we conducted MR analysis using GWAS data for 14 medication categories and the GWAS data of migraine with its subtypes. MR is based on three fundamental assumptions: (i) the relevance assumption, which requires a strong association between instrumental variables (IVs) and the exposure; (ii) the independence assumption, stating that IVs influence the outcome solely through the exposure; (iii) the exclusion restriction assumption, which dictates that IVs should not have a direct impact on the outcome (Emdin et al. 2017). We selected IVs based on stringent *p* value thresholds (SNPs with *p* < 5e‐08) and employed a clumping process to reduce the effects of high‐signal SNPs resulting from linkage disequilibrium using a window of 10,000 and an *R* ^2^ threshold of 0.001. To ensure robust statistical linkage between SNPs and exposure, we calculated the F‐statistic, excluding SNPs with values less than 10 (Burgess et al. 2013). The specific formula used for these calculations is as follows (Kurilshikov et al. 2021):

$$F=\frac{R^{2}\left(N-2\right)}{\left(1-R^{2}\right)},$$

where *N* and *R* ^2^ refer to the sample size and the variance explained by IVs, respectively.

In the 23 medication‐use categories, we found that Vasodilators used in cardiac diseases, Antihypertensives, Opioids, and Antidepressants lacked sufficient valid instrumental variables. Therefore, we identified valid instrumental variables from the remaining 19 drug classes. Moreover, we excluded five ATC (Anatomical Therapeutic Chemical) classes directly associated with migraine, which we identified from the drug database. These excluded classes include Salicylic acid and derivatives, Anilides, Antimigraine preparations, Beta blocking agents, and Calcium channel blockers. Consequently, we obtained GWAS data for 14 drug classes, which were used in the MR analysis, as detailed in Table S2. We primarily utilized the inverse variance weighted (IVW) method, which was chosen for its efficacy in scenarios with valid IVs and its ability to handle heterogeneity in variant‐specific causal estimates. For significant findings from the IVW analysis, additional MR methods including weighted median, MR‒Egger, simple mode, and weighted mode, were employed (Verbanck et al. 2018). Heterogeneity in the results was assessed using IVW and MR‐Egger, along with Cochran's *Q* test, and *p*‐value < 0.05 indicated the robust results. These analyses were conducted using the R packages, which include TwoSampleMR, MR‐PRESSO, and ieugwasr.

### 2.3 TWAS and Cross‐Reference of Three Drug Databases

TWAS was conducted within the OTTERS framework (Dai et al. 2023). OTTERS involves two key stages. Stage I: we employed a variety of methods to deduce cis‐eQTL weights from summary‐level cis‐eQTL data alongside an external linkage disequilibrium (LD) reference panel. These methods included P+T (*p*‐value thresholding with LD clumping) (Purcell et al. 2009), lassosum (Mak et al. 2017), SDPR (Zeng and Zhou 2017; Zhou and Zhao 2021), and PRS‐CS (Ge et al. 2019). Stage II: Using the derived cis‐eQTL weights, we estimated the GReX for each gene and then perform gene‐based association analyses within the GWAS dataset. We generated a set of TWAS p‐values using each of the trained models and consolidated them into a single composite test result using the Aggregated Cauchy Association Test (ACAT‐O) method (Liu et al. 2019), and the resulting *p*‐values are referred to as OTTERS *p*‐values. To ensure the robustness of our results, we applied false discovery rate (FDR) correction to the p‐values. An FDR‐adjusted *p*‐value < 0.05 was considered statistically significant for associations between the gene and migraine or its subtypes.

Following the contraindicated medications determined by our MR analyses, we also directly extracted their targeted genes according to three drug databases: DrugBank (Wishart et al. 2018), Ruiz et al. (2021), and ChEMBL (Gaulton et al. 2017). Then, we intersected all these target genes with the genes identified in TWAS to further obtain the target genes of contraindicated medications related to migraine and its subtypes.

### 2.4 INTACT

To further and effectively confirm the targeted genes of contraindicated medications, we used the INTACT framework (Okamoto et al. 2023), a novel method that combines TWAS with colocalization. This approach integrates probabilistic evidence from these different types of analyses to infer the putative pathogenic genes. Bayesian colocalization analysis was conducted using the “coloc” package, for each target gene, SNPs within a ±500 kb range of eQTLs were analyzed, with each eQTL receiving a separate evaluation and particular emphasis, then prior probability for the five hypothesis (PP.H0‐PP.H4) was generated, and the integrated PP was calculated using the formula PP.H4/(PP.H3+PP.H4) (Giambartolomei et al. 2014). Subsequently, under the usage of INTACT, we combined the Z values from the TWAS with the integrated PP obtained, resulting in a new colocalization probability, the INTACT probability. A significant association was identified when the INTACT probability exceeded a threshold of 0.8.

### 2.5 SMR and HEIDI

To further validate the target genes of contraindicated drugs identified, we introduced SMR analysis, with HEIDI differentiating whether the associations between targeted genes and migraine were because of a shared genetic variant or genetic linkage (Wu et al. 2018). The significance threshold for the SMR was set at a *p*‐value of less than 0.05 and a *p*‐value greater than 0.01 in the HEIDI suggested that the associations were not influenced by linkage disequilibrium. All analyses were performed using SMR software (version 1.03).

### 2.6 Brain tissue TWAS to Validate Disease‐Associated Tissue‐Specific Expression of Hub Genes

To assess whether hub genes show disease related expression in the brain, we performed transcriptome wide association studies using the FUSION framework (Gusev et al. 2016). FUSION imputes genetically regulated gene expression from GWAS summary statistics and evaluates gene level associations with complex traits and diseases. We used GTEx pretrained eQTL weights for 13 brain regions: amygdala, anterior cingulate cortex BA24, caudate in the basal ganglia, cerebellar hemisphere, Cerebellum, cortex, frontal cortex BA9, hippocampus, hypothalamus, nucleus accumbens in the basal ganglia, putamen in the basal ganglia, spinal cord cervical C1, and substantia nigra. Analyses covered migraine and related subtypes and were executed on Linux with FUSION.

## 3 Results

### 3.1 Mendelian Randomization

**FIGURE 2:**
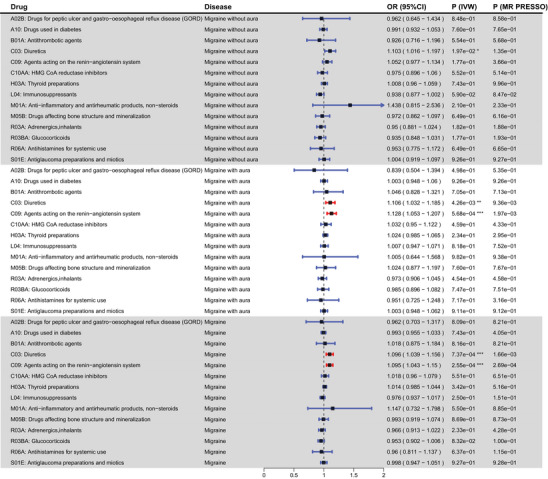
Forest plot of MR analysis for 14 medication‐use categories and migraine and its two subtypes.

We examined the causal relationships between 14 medication categories and migraine and its subtypes through MR. Four significant causal associations were identified, all of which are related to an increased risk and the main analysis (IVW) results are as follows: diuretics (OR (95% CI) = 1.106 (1.032–1.185), *p* = 4.26×10^−3^) and agents acting on the renin‐angiotensin system (OR (95% CI) = 1.128 (1.053–1.207), *p* = 5.68×10^−4^) increased the risk of migraine with aura; diuretics (OR (95% CI) = 1.096 (1.039−1.156), *p* = 7.37×10^−4^) and agents that act on the renin‐angiotensin system (OR (95% CI) = 1.095 (1.043−1.15), *p* = 2.55×10^−4^) also exhibited a heightened propensity for contributing to migraine (Table S3 and Figure 2). The results mentioned above are consistent among the IVW findings and the four additional methods. They also passed the horizontal pleiotropy test (using the MR‐Egger test, with *p* > 0.05) and the MR‐PRESSO test (*p* < 0.05). Further details on the results from the remaining MR analysis methods and heterogeneity tests can be found in Table S3. Concerning migraine without aura, we did not find any medications with latent causality.

### 3.2 TWAS

**FIGURE 3:**
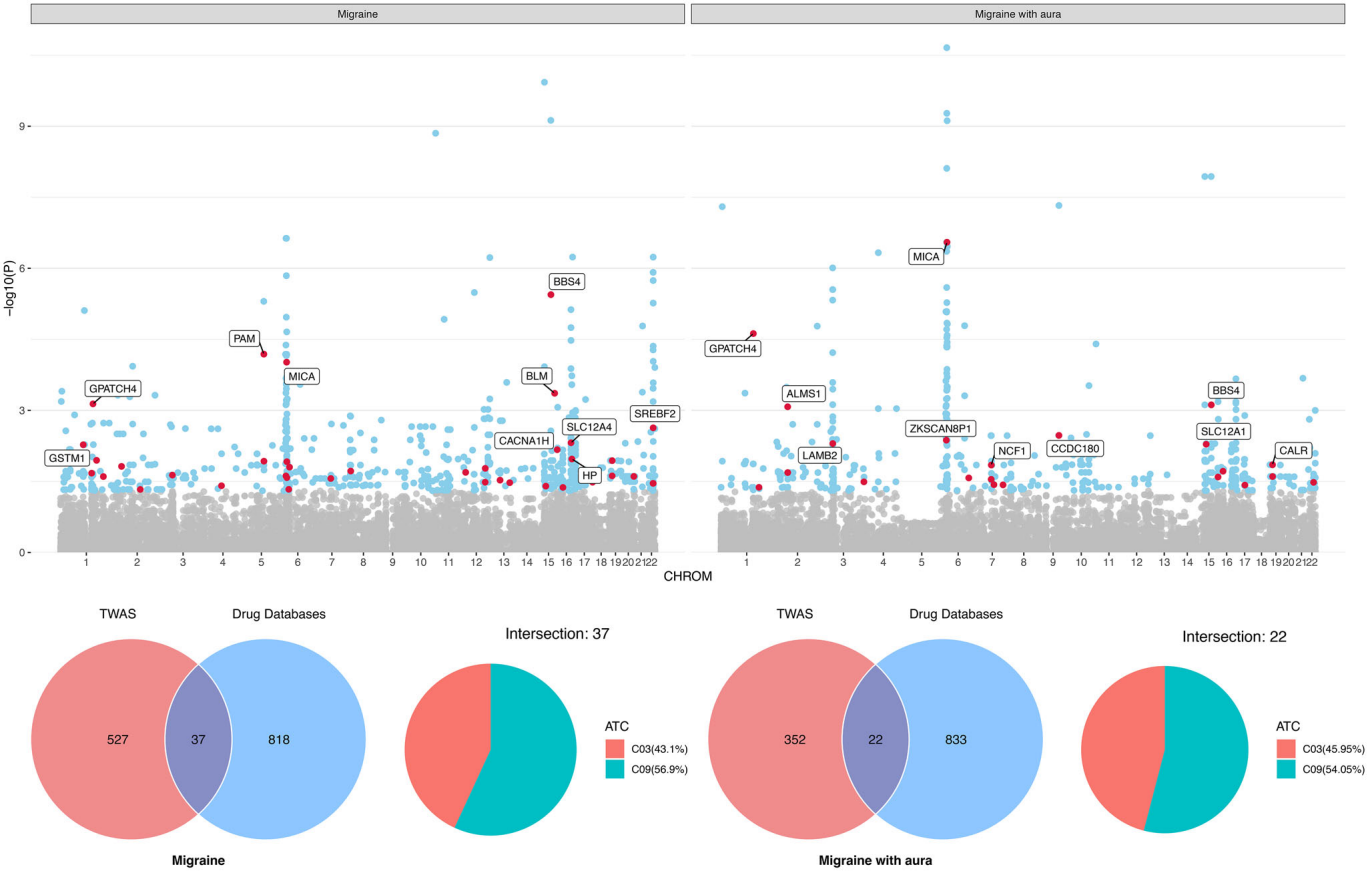
Manhattan plot, Venn diagram, and pie chart of the TWAS. (A) Manhattan plot of the TWAS results. (gray: FDR_TWAS_ > 0.05; blue: FDR_TWAS_ < 0.05; red indicates the target genes within the drug databases with FDR_TWAS_ < 0.05). (B) Venn diagram (left) and pie chart (right) of significant genes identified by the TWAS (FDR < 0.05) and target genes from the drug database.

TWAS enabled the identification of 564 genes associated with migraine and 374 genes linked to migraine with aura (FDR < 0.05) (Figure 3A, Table S4). Among these genes, the five most significantly associated with migraine were *BBS4* (*p* = 3.60×10^−6^), *PAM* (*p* = 6.50×10^−5^), *MICA* (*p* = 9.60×10^−5^), *BLM* (*p* = 4.35×10^−4^), and *GPATCH4* (*p* = 7.29×10^−4^). For migraine with aura, *MICA* (*p* = 2.82×10^−7^), *GPATCH4* (*p* = 2.39×10^−5^), *BBS4* (*p* = 7.64×10^−4^), *ALMS1* (*p* = 8.40×10^−4^), and CCDC180 (*p* = 3.38×10^−3^) were the five most significant ones.

For the two contraindicated medications for migraine and migraine with aura, we extracted a total of 855 targeted genes by cross‐referencing the three drug databases (Figure 3B). Then, an intersection analysis was carried out, resulting in 37 targeted genes for contraindicated drugs related to migraine and 22 for migraine with aura for subsequent analysis (Table S5, Figure 3B).

### 3.3 INTACT

**FIGURE 4:**
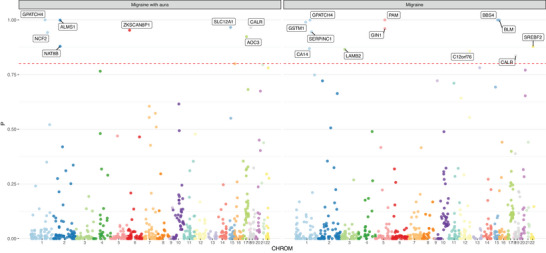
Manhattan plot of INTACT results. The *x*‐axis represents chromosomal positions, and the *y*‐axis represents colocalization probabilities obtained from INTACT. The red line represents the set threshold = 0.8.

Figure 4 and Tables S6 and S7 show the results of INTACT. For migraine, out of the 37 targeted genes of contraindicated medications, 12 were validated. Among them, the genes with the highest INTACT values are *PAM* (INTACT = 0.999), *BBS4* (INTACT = 0.999) and *GPATCH4* (INTACT = 0.999). For migraine with aura, out of 22 genes, 8 showed strong evidence of colocalization. The most prominent gene was *GPATCH4* (INTACT = 0.999), followed by *ALMS1* (INTACT = 0.998). It is noteworthy that GPATCH4 exhibited the most significant colocalization evidence for both phenotypes. Genes with significant colocalization evidence were used for subsequent analysis.

### 3.4 SMR and HEIDI

Using SMR and HEIDI, we ultimately identified 5 genes causally associated with migraine with aura from the 8 targeted genes of contraindicated drugs that passed INTACT validation, among which three are common targets of Diuretics and Agents acting on the renin‐angiotensin system, and are associated with an increased risk of migraine with aura: *ALMS1* (OR (95% CI) = 1.14 (1.051−1.237), *P*_SMR_ = 1.61×10^−3^), *GPATCH4* (OR (95% CI) = 1.783 (1.242−2.56), *P*_SMR_ = 1.71×10^−3^), and *ZKSCAN8P1* (OR (95% CI) = 1.409 (1.19−1.668), *P*_SMR_ = 7.12×10^−5^). The target NCF2, associated with Agents acting on the renin‐angiotensin system, is linked to a reduced risk of migraine with aura (OR (95% CI) = 0.674 (0.538−0.843), *P*_SMR_ = 5.60×10^−4^). Similarly, the shared target SLC12A1 of both Diuretics and Agents acting on the renin‐angiotensin system is also connected to a decreased risk of migraine with aura (OR (95% CI) = 0.954 (0.927−0.981) and *P*_SMR_ = 1.08×10^−3^).

**FIGURE 5:**
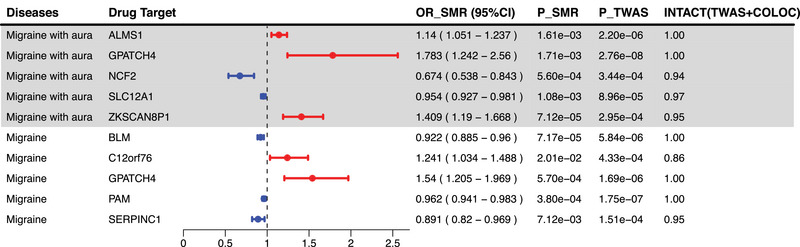
The results of SMR and HEIDI for the targeted genes of contraindicated medications for migraine and migraine with aura.

For migraine, we ultimately identified 5 out of the 12 targeted genes of contraindicated medications as being causally associated with it. Among these, two common targets of Diuretics and Agents acting on the renin‐angiotensin system are associated with a reduced risk of migraine: *BLM* (OR (95% CI) = 0.922 (0.885−0.96), *P*_SMR_ = 7.17×10^−5^), *PAM* (OR (95% CI) = 0.962 (0.941−0.983), *P*_SMR_ = 3.80×10^−4^), and *SERPINC1* (OR(95% CI) = 0.891 (0.82−0.969), *P*_SMR_ = 7.12×10^−3^). The common targets *C12orf76* (OR (95% CI) = 1.241 (1.034−1.488), *P*_SMR_ = 2.01×10^−2^) and *GPATCH4* (OR (95% CI) = 1.54 (1.205−1.969), and *P*_SMR_ = 5.70×10^−4^) of both Diuretics and Agents acting on the renin‐angiotensin system are associated with a reduced risk of migraine (Figure 5, Table 1).

**TABLE 1:** The targeted gene‐contraindicated drug pairs that are causally associated with migraine and migraine with aura.

Diseases	Symbol	Drug	*P*_TWAS (FDR)_	INTACT	*OR*_SMR_	*P*_SMR_	*P*_HEIDI_
Migraine with aura	ALMS1	C03: diuretics; C09: agents acting on the renin‐angiotensin system	0.000840762	0.998834	1.140077	0.001608	0.228028
Migraine with aura	GPATCH4	C03: diuretics; C09: agents acting on the renin‐angiotensin system	2.39E‐05	0.999969	1.783197	0.001711	0.636482
Migraine with aura	NCF2	C09: agents acting on the renin‐angiotensin system	0.042245138	0.942408	0.673785	0.00056	0.423232
Migraine with aura	SLC12A1	C03: diuretics; C09: Agents acting on the renin‐angiotensin system	0.005194482	0.965651	0.95391	0.001084	0.424451
Migraine with aura	ZKSCAN8P1	C03: diuretics; C09: agents acting on the renin‐angiotensin system	0.004268762	0.952731	1.408602	7.12E‐05	0.13765
Migraine	BLM	C09: agents acting on the renin‐angiotensin system	0.000435782	0.998024	0.921657	7.17E‐05	0.338013
Migraine	C12orf76	C03: diuretics; C09: agents acting on the renin‐angiotensin system	0.01678485	0.857452	1.240657	0.02005	0.334854
Migraine	GPATCH4	C03: diuretics; C09: agents acting on the renin‐angiotensin system	0.000729437	0.999232	1.539995	0.00057	0.984173
Migraine	PAM	C09: agents acting on the renin‐angiotensin system	6.50E‐05	0.999867	0.961537	0.00038	0.443699
Migraine	SERPINC1	C03: diuretics; C09: agents acting on the renin‐angiotensin system	0.011351606	0.945034	0.891246	0.007118	0.266813

### 3.5 Brain tissue TWAS to Validate Disease‐Associated Tissue‐Specific Expression of Hub Genes

To validate the relevance of our hub genes to migraine and its aura subtype in brain tissues, we conducted TWAS using the FUSION framework by integrating eQTL reference weights from 13 GTEx brain regions with GWAS summary statistics for migraine and migraine with aura (Table S8). In migraine, SERPINC1, ZKSCAN8P1, C12orf76, and PAM showed significant associations across brain regions (*p* < 0.05); notably, SERPINC1 was significant in 12 of the 13 regions (all except Frontal cortex BA9), and the strongest signal was observed for PAM in the Putamen (basal ganglia) (*p* = 1.05 × 10^−4^). For migraine with aura, SERPINC1, ALMS1, ZKSCAN8P1, PAM, and NCF2 were significant (*p* < 0.05); SERPINC1 was significant in nine regions, with the most pronounced association in the Nucleus accumbens (basal ganglia) (*p* = 0.014).

## 4 Discussion

The objective was to identify drugs with contraindications and the targeted genes associated with an increased risk of migraine and its subtypes, with the aim of deepening our understanding of novel preventive strategies. To achieve the objective, we initially conducted MR analyses using GWAS summary statistics for 14 medication categories and migraine and its subtypes, revealing that both diuretics and agents that act on the renin‐angiotensin system are positively associated with migraine and migraine with aura. Then we employed the OTTERS framework for TWAS to identify 564 and 374 genes that are related to migraine and migraine with aura, respectively. By intersecting these genes with the 855 drug target genes extracted from three drug databases, we were able to identify 37 migraine‐related genes and 22 migraine‐with‐aura‐related targeted genes of the contraindicated drugs. The results of the SMR, colocalization, and INTACT sensitivity analyses provided further validation of BLM, C12orf76, GPATCH4, PAM, SERPINC1 as contraindicated drugs’ target genes for migraine, and ALMS1, GPATCH4, NCF2, SLC12A1, ZKSCAN8P1 for migraine with aura. Of particular note, GPATCH4, a target common to diuretics and agents acting on the renin angiotensin system was significantly associated with increased risk of both migraine and migraine with aura. At the brain‐tissue level, we further validated hub gene associations: in migraine, SERPINC1, ZKSCAN8P1, C12orf76, and PAM were significant across brain regions; in migraine with aura, SERPINC1, ALMS1, ZKSCAN8P1, PAM, and NCF2 were significant.

*PAM*, a target of agents that act on the renin‐angiotensin system, has been demonstrated to activate mGlu2 (metabotropic glutamate receptor 2). mGlu2 is predominantly expressed in the forebrain and serves as a presynaptic modulator of glutamate, and it has been identified as a potential target for the treatment of anxiety disorders, schizophrenia, and migraine (Blanco et al. 2017). Agents that act on the renin‐angiotensin system may inhibit this process, thereby elevating the risk of migraine. For *SERPINC1*, it has been demonstrated as a serine protease inhibitor known as antithrombin III (ATIII), a common target of diuretics and agents that act on the renin‐angiotensin system (Natae et al. 2021). Consequently, we hypothesize that these drugs may increase the risk of migraine through this pathway, which may increase the risk of migraine by affecting this process (Bianchi et al. 1996). Knockdown of *GPATCH4* leads to reduced cell viability and is associated with cellular aging (Kodera et al. 2024), which appears to be related to oxidative stress, making itself a potential cause of migraine (Fila et al. 2023). Beyond effects on cell survival and senescence, GPATCH4 is a nucleolar G‐patch cofactor that partners with the RNA helicase DHX15 to support snRNA/rRNA processing and ribosome biogenesis (Kanwal et al. 2024). Disruption of this axis can trigger nucleolar stress and stress‐responsive translational reprogramming, consistent with reports that GPATCH4 maintains nucleolar morphology and cell viability (Kodera et al. 2024; Maehama et al. 2023). In migraine‐relevant neurovascular and immune contexts, such changes could lower the threshold for trigeminovascular activation and amplify oxidative/inflammatory signaling—processes implicated in migraine pathophysiology (Ferrari et al. 2022; Borkum 2016; Shatillo et al. 2013). By contrast, C12orf76 remains poorly characterized. Public resources annotate it as a predicted membrane‐associated protein, with transcript evidence across multiple brain regions, supporting a plausible interface with neurovascular or immune signaling relevant to migraine biology (HPA; GTEx) (Uhlen et al. 2015; Sjostedt et al. 2020; Carithers and Moore 2015). In addition, the broader 12q24 region has been implicated in migraine‐related genetics. For example, cross‐phenotype GWAS mapping reveals a strong shared signal at 12q24 between migraine and ischemic stroke, and family‐based linkage studies have identified the 12q24.2–q24.3 subregion in association with migraine. However, precise gene assignment at this locus remains uncertain (Malik et al. 2015). Overall, C12orf76 remains poorly characterized but shows brain expression and predicted membrane localization with 12q24 regional signals, making it a plausible yet provisional neurovascular/immune interface for migraine.

*NCF2*, also known as *NOXA2*, is mainly involved in cellular oxidative stress (Yan et al. 2023). Given that both inflammation and oxidative stress are linked to migraine pathogenesis (Jimenez‐Jimenez et al. 2024) and increased oxidative stress has been specifically observed in patients with migraine with aura (Tuncel et al. 2008), it is plausible that agents that act on the renin‐angiotensin system can increase migraine risk by the approaches. Additionally, mutations in the *ALMS1* gene can cause Alström syndrome, which is characterized by progressive metabolic changes, including hypertension and chronic kidney disease (Jaykumar et al. 2018), and it is noteworthy that hypertension, particularly when chronic and uncontrolled, is associated with increased incidences of both aura and non‐aura migraines (Gardener et al. 2016). It can thus be postulated that the diuretics and agents in question contribute to the elevated risk of migraine by influencing these physiological processes, integrating the molecular and clinical observations into a coherent mechanism of disease exacerbation.

Intriguingly, our findings appear to contradict established strategies for migraine prevention and treatment that have demonstrated efficacy in clinical studies and trials, such as angiotensin receptor blockers (e.g., candesartan) (Schrader et al. 2001; Tronvik et al. 2003; Garcia‐Azorin et al. 2024; Camarda et al. 2003; Ikeda et al. 2017; Diener et al. 2009; Silberstein et al. 2012). This may reflect the combined effects of dose and duration. In randomized crossover trials, short‐term prophylaxis within a defined evaluation window has demonstrated clinical benefit; for instance, candesartan at 16 mg/day and lisinopril at 10–20 mg/day were shown to reduce migraine or headache days over approximately 12 weeks, indicating that both dosage and duration of exposure are clinically relevant (Diener et al. 2016). Conversely, with prolonged exposure in routine care, the net effect may shift; experience with other drug classes shows that higher or more frequent use can worsen headache burden, making a time‐ and dose‐dependent, potentially biphasic pattern plausible but unproven (Gosalia et al. 2024). Additionally, patient‐specific factors may contribute to this discrepancy. Genetic variation within the RAAS has been linked to migraine susceptibility in some populations (e.g., the ACE insertion/deletion polymorphism) and has been examined at AGTR1 A1166C and AGT M235T, although results are heterogeneous. Pharmacogenomic studies likewise document variability in responses to ACE inhibitors and ARBs; notably, the ACE I/D polymorphism has not consistently predicted treatment benefit in clinical settings (Schurks et al. 2010; Sudershan et al. 2023; Ohmichi et al. 1997; Perna et al. 2000; Tronvik et al. 2008). In summary, these factors may account for the risk association inferred from our genetic evidence; confirmation will require agent‐ and time‐resolved clinical and real‐world studies incorporating stratified analyses across relevant populations.

Additionally, in real‐world care many patients take more than one medication class at the same time, and those combinations can interact to alter migraine risk and course. Observational and longitudinal data support this: frequent or combined use of acute medications is linked to higher headache burden and progression to migraine (Ferrari et al. 2018; Ashina et al. 2023). The risk is not uniform across classes combination analgesics (especially those containing opioids or barbiturates) carry the highest risk, single‐ingredient analgesics are intermediate, and triptans/ergots appear lower (Ashina et al. 2023; Vandenbussche et al. 2018; Ljubisavljevic et al. 2023). Accordingly, future research on medication‐related migraine risk should incorporate specific agents and common co‐prescribing patterns, explicitly test additive and interaction effects, and employ stratified analyses, such as those based on baseline attack frequency and the temporal course of exposure, to better reflect real‐world treatment practices.

Throughout the study, we need to re‐emphasize the highlights of the study. Over the past decade, GWAS have revolutionized the field of complex disease genetics, providing a wealth of compelling associations for human complex traits and diseases (Tam et al. 2019). MR analysis can use GWAS and utilize genetic variations as IV to infer causal relationships between exposures and outcomes, overcoming confounding factors such as behavioral and environmental influences, and providing robust evidence for the causal relationship between risk factors and diseases (Davey Smith and Hemani 2014; Burgess et al. 2012; Smith and Ebrahim 2004). On the other hand, the TWAS is a valuable analysis strategy for identifying genes that influence complex traits and diseases through the genetic regulation of gene expression. It has been widely applied in identifying disease‐associated genes (Mancuso et al. 2017; Gusev et al. 2018; Mancuso et al. 2018; Wainberg et al. 2019; Strunz et al. 2020). LD may lead to false positive associations identified through GWAS, whereas TWAS can provide a higher proportion of true positive results and is less affected by LD compared to GWAS (Li et al. 2021). OTTERS is a powerful TWAS framework that, compared to other TWAS frameworks, has a significant advantage in handling summary‐level data with relatively large sample sizes (Dai et al. 2023). INTACT methods surpass colocalization‐centric approaches in identifying genes with greater efficacy while effectively controlling for Type I errors typically caused by LD in TWAS methods (Okamoto et al. 2023).

It is also essential to recognize the constraints of our study. Firstly, our analyses primarily involve European‐ancestry cohorts, which may limit applicability to other populations due to ancestry‐specific allele‐frequency spectra and linkage disequilibrium patterns. Accordingly, we recommend broad trans‐ethnic studies and validation prior to clinical translation. Secondly, the mechanisms by which diuretics and agents acting on the renin‐angiotensin system affect the risk of migraines require validation through additional clinical studies and experimental evidence. Third, we limited migraine subtyping to with or without aura because this classification, like overall migraine, is derived from the same large cohort GWAS, ensuring comparability across datasets. We acknowledge that other clinically significant subtypes, such as chronic migraine and medication‐overuse headache, may differ in disease burden, medication exposure, and the uptake of preventive treatments. Therefore, broader subtype‐focused studies are needed to determine whether the risk medications and prioritized targets identified here extend to a wider range of migraine subtypes. Last but not least, despite the broad scope of our study that encompassed a wide range of plasma genes and identified potential target genes associated with contraindicated medications, the absence of certain genetic instruments may have led to the unintentional omission of additional candidate genes.

In conclusion, we employed a multi‐faceted approach, integrating MR, TWAS within the OTTERS framework, SMR, and colocalization, to identify contraindicated drugs and their target genes. Diuretics and agents acting on the renin‐angiotensin system were identified as potential contraindicated medications, and their target genes, including ALMS1, GPATCH4, NCF2, SLC12A1, ZKSCAN8P1, BLM, C12orf76, PAM, and SERPINC1, were identified as the pivotal mechanisms. The findings presented herein not only elucidate the genetic basis of migraine but also provide evidence of potential contraindications, which will contribute to the development of more effective and targeted prevention strategies.

## Author Contributions

All the authors made significant contributions to this work and approved the final manuscript. Concept and design: NW, WXL, ZFW, WZL, CGH, and HBZ. Data curation: HBZ, NW, WXL, ZFW, and ZYY. Analysis and interpretation of data: NW, HBZ, WXL, ZFW and WZL. Computational resources and support: NW, ZYZ, WXL, ZXX, CGH, and XTJ. Writing of the original draft and reviews: XTJ, WXL, ZFW, HBZ, ZYY, and WZL. Editing draft and reviews: NW, HBZ, ZYZ, WXL, ZFW, and XTJ.

## Conflicts of Interest

The authors declare that there is no conflict of interest.

## Funding

The authors have nothing to report.

## Ethics Statement

No additional ethics approval was needed because all data in the study was previously collected, analyzed, and published.

## Supporting information




**Table S1**: GWAS summary statistics for medication‐used categories and migraine with its subtypes, and eQTL summary data.
**Table S2**: Medications directly related to migraine and its subtypes.
**Table S3**: MR results and supplementary analyses for medication‐used categories and migraine with its subtypes.
**Table S4**: Results of TWAS identifying genes associated with migraine and its subtypes.
**Table S5**: The drug targets corresponding to drugs with side‐effects for migraine and its subtypes.
**Table S6**: Colocalization further confirming contraindicated drug target genes associated with migraine and its subtypes.
**Table S7**: Results of INTACT integrating TWAS and colocalization.
**Table S8**: Brain‐tissue TWAS validating tissue‐specific associations between hub genes and migraine and its subtypes.

## Data Availability

Publicly available datasets were analyzed in this study. This data can be found in Supplementary S1.
